# Knowledge and Practice Regarding Stroke Prevention and Associated Factors Among Hypertensive Patients at Selected Public Hospitals in Addis Ababa, Ethiopia: A Cross‐Sectional Study

**DOI:** 10.1002/hsr2.73268

**Published:** 2026-09-17

**Authors:** Hana Shafi Amde, Alfoalem Araba Abiye, Negalign Getahun Dinegde, Emebet Berhane Woldemariam

**Affiliations:** ^1^ Addis Continental Institute of Public Health Addis Ababa Ethiopia; ^2^ Department of Pharmacology and Clinical Pharmacy School of Pharmacy, College of Health Sciences, Addis Ababa University Addis Ababa Ethiopia; ^3^ School of Nursing and Midwifery, College of Health Sciences, Addis Ababa University Addis Ababa Ethiopia

**Keywords:** Ethiopia, health‐promoting lifestyle, hypertension, knowledge, practice, stroke prevention

## Abstract

**Background and Aims:**

Stroke is a leading cause of death and disability worldwide, and hypertension is its strongest modifiable risk factor. The protection that treatment offers, however, depends on whether patients recognize their own risk and sustain preventive behavior. This has not been examined in Addis Ababa, where patients have comparatively good access to specialist follow‐up. We assessed knowledge and practice regarding stroke prevention at two public hospitals in the city, and the characteristics associated with each.

**Methods:**

An institution‐based cross‐sectional study was conducted from 1 February to 20 August 2022 among 283 hypertensive adults attending cardiac follow‐up at two federal teaching hospitals. Knowledge was measured with the 32‐item Stroke Prevention Knowledge Questionnaire and dichotomized at ≥ 60% correct; practice was measured with the 52‐item Health‐Promoting Lifestyle Profile II (HPLP‐II) and analyzed as a continuous score. Binary logistic and multivariable linear regression were used respectively; all tests were two‐sided with *α* = 0.05.

**Results:**

Seventy‐seven participants (27.2%) had good overall knowledge; 137 (48.4%), 101 (35.7%) and 59 (20.8%) reached the domain thresholds for prevention measures, warning signs and risk factors, and 110 (38.9%) named no warning sign. Educational attainment (above Grade 12 vs. no formal schooling—adjusted odds ratio: 50.43, 95% confidence interval [CI]: 10.98–231.53), religion and family history of stroke were independently associated with good knowledge. The mean HPLP‐II score was 136.97 ± 26.71, within the “good” band, but physical activity scored lowest. Educational attainment, widowhood and good knowledge (adjusted mean difference: 13.20, 95% CI: 6.81–19.60) were independently associated with practice.

**Conclusion:**

Knowledge of stroke prevention was poor despite favorable lifestyle scores, and educational attainment was associated with both. Attendance at a specialist clinic is not evidence that a patient understands their risk; counseling should verify comprehension rather than assume it.

## 1 Introduction

Stroke is among the leading causes of death and long‐term neurological disability globally, and its burden is concentrated in low‐ and middle‐income countries (LMICs) [1]. It arises from acute vascular injury to the brain, presenting as ischaemic infarction, intracerebral haemorrhage or subarachnoid haemorrhage [2]. Raised blood pressure, elevated body mass index (BMI), hyperglycaemia, smoking and air pollution are the principal modifiable contributors [3], with hypertension the single most important among them [4]. Global Burden of Disease estimates for 2019 put prevalent cases at 101.5 million and incident cases at 12.2 million, with 143 million disability‐adjusted life years lost; ischaemic stroke accounted for 3.3 million deaths, intracerebral haemorrhage 2.9 million and subarachnoid haemorrhage 0.4 million [5]. Over the same period the number of adults with hypertension worldwide doubled, with the increase concentrated in LMICs and treatment coverage lagging furthest behind in sub‐Saharan Africa [6].

Because most of this burden is attributable to modifiable exposures, prevention rather than acute treatment offers the greater return, particularly where acute stroke services are scarce and outcomes after stroke are correspondingly poor [7, 8, 9]. Population‐level measures such as salt reduction, tobacco control and promotion of physical activity constitute primordial prevention, while control of hypertension, diabetes and dyslipidaemia constitutes primary prevention [8]. Sustained lowering of blood pressure substantially reduces the risk of both first and recurrent stroke, and the size of that reduction tracks the degree of blood pressure control achieved [10, 11].

In Ethiopia, stroke accounts for approximately 6.23% of all deaths [12], and hypertension, diabetes and atrial fibrillation together underlie roughly half of all stroke events [13]. Acute stroke care remains concentrated in a small number of tertiary centers, with limited imaging, thrombolysis and rehabilitation capacity [14], which places the practical burden of risk reduction on outpatient chronic‐disease clinics and on patients themselves. Preventive benefit therefore depends on whether patients can recognize their own risk factors, identify warning signs, and maintain the behaviors that lower risk [15, 16]. Studies across Ethiopia have repeatedly found this recognition to be incomplete: roughly a quarter of hypertensive patients at Debre Tabor General Hospital had good preventive knowledge [17], and substantial proportions elsewhere could not name a single major risk factor or warning sign [18].

What remains unknown is how knowledge and preventive behavior relate to one another in an urban tertiary setting. Previous Ethiopian studies have measured knowledge [17, 18, 19, 20, 21], and a smaller number have measured preventive practice [20, 22], but they have generally treated the two separately, have been conducted in regional rather than metropolitan hospitals, and have used ad hoc practice checklists rather than a validated lifestyle instrument. Whether hypertensive patients in Addis Ababa, who have comparatively good access to specialist cardiac follow‐up, show the same knowledge deficit, and whether that deficit corresponds to poorer health‐promoting behavior in the same individuals, has not been established.

This study therefore had three objectives: (i) to determine the level of knowledge regarding stroke prevention, warning signs and risk factors among hypertensive patients attending two public hospitals in Addis Ababa; (ii) to determine the level of stroke prevention practice in the same population using a validated health‐promoting lifestyle instrument; and (iii) as a co‐primary objective, to identify the sociodemographic and clinical characteristics associated with each outcome. Objectives (i) and (ii) were pre‐specified as descriptive and objective (iii) as analytic.

## 2 Materials and Methods

The study is reported in accordance with the Strengthening the Reporting of Observational Studies in Epidemiology (STROBE) statement for cross‐sectional studies [23]; the completed checklist is provided as Supporting Information S1: File S1. Statistical reporting follows the SAMPL guidelines [24] and, where transferable to an observational design, the recommendations of Assel and colleagues [25].

### 2.1 Study Setting

The study was conducted at St. Paul's Hospital Millennium Medical College (SPHMMC) and St. Peter's Specialized Hospital (SPSH), both federal teaching hospitals in Addis Ababa. SPHMMC is a 400‐bed government facility serving more than 400,000 patients annually across outpatient, emergency and inpatient services. SPSH, established in 1963 as a national tuberculosis center, now employs approximately 500 staff, of whom 250 are health professionals, and provides internal medicine, dermatology, ophthalmology, paediatric, maternal and child health, mental health and dental services alongside its tuberculosis and TB/HIV work. SPSH operates a cardiac catheterization laboratory, echocardiography, a cardiac critical care unit, and outpatient and inpatient cardiac services in collaboration with SPHMMC. Both hospitals draw referrals nationally, so their cardiac clinics serve a population wider than the capital.

### 2.2 Study Design and Period

An institution‐based cross‐sectional study was conducted between 1 February and 20 August 2022 among hypertensive patients attending routine follow‐up at the cardiac clinics of SPHMMC and SPSH.

### 2.3 Populations

The source population comprised all hypertensive patients on follow‐up at the SPHMMC and SPSH cardiac clinics. The study population comprised those attending during the data collection period who met the eligibility criteria.

### 2.4 Eligibility Criteria

**Inclusion:** adults aged ≥ 18 years with a documented diagnosis of hypertension, attending a scheduled appointment at either cardiac clinic during the study period, and willing to give written informed consent.

**Exclusion:** prior transient ischaemic attack or stroke; critical illness; severe mental illness; or inability to respond independently to the interview.

### 2.5 Sample Size

Sample size was estimated using the single population proportion formula:

$$n=\left[{Z_{\alpha /2}}^{2}\times p\left(1-p\right)\right]/d^{2},$$

where *n* is the required sample size, *p* the assumed proportion with good knowledge of stroke prevention, *d* the margin of error, and *Z*_α/2_ the standard normal deviate for the chosen confidence level. With *p* = 0.25, the proportion with good stroke prevention knowledge reported from Debre Tabor General Hospital [17]; *d* = 0.05; and *Z*_α/2_ = 1.96 for a 95% confidence level, this gave *n* = 288. Allowing for 5% non‐response, the target sample was 302.

### 2.6 Sampling Procedure

**Figure 1:**
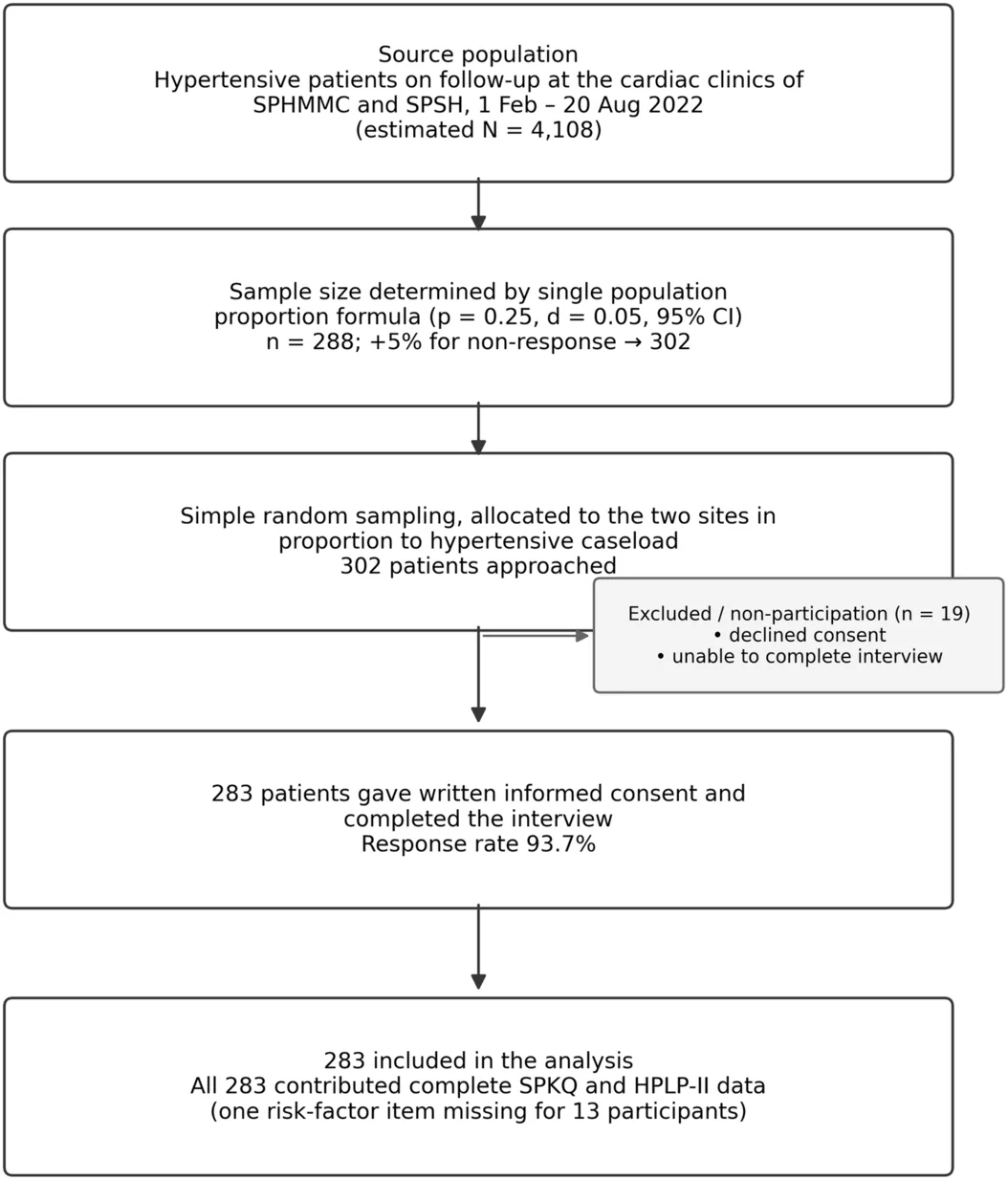
Participant flow and sampling, cardiac follow‐up clinics of St. Paul's Hospital Millennium Medical College and St. Peter's Specialized Hospital, Addis Ababa, Ethiopia, 1 February to 20 August 2022. The 302 sampled participants were allocated between the two sites in proportion to each site's hypertensive caseload, from a combined estimated source population of 4108 patients.

Participants were selected by simple random sampling from the two hospitals, with the 302 allocations distributed proportionally to each site's hypertensive caseload, using

$$n_{\mathrm{site}}=n_{\mathrm{total}}\times 1\left(N_{\mathrm{site}}/N_{\mathrm{total}}\right),$$

where *N*_site_ is the number of hypertensive patients on follow‐up at that site and *N*_total_ the combined estimated caseload of 4108 patients. The sampling process and participant flow are shown in Figure 1.

### 2.7 Variables

#### 2.7.1 Outcome Variables

- *Knowledge regarding stroke prevention*, analyzed as a binary variable (good vs. poor), derived by dichotomizing the total 32‐item Stroke Prevention Knowledge Questionnaire (SPKQ) score at ≥ 60% correct.
- *Practice regarding stroke prevention*, analyzed as a continuous variable, the total Health‐Promoting Lifestyle Profile II (HPLP‐II) summed score (possible range: 52–208).

#### 2.7.2 Explanatory Variables

Sociodemographic: age, sex, marital status, educational status, religion and monthly income. Clinical: duration of hypertension since diagnosis, BMI, and family history of stroke. Blood pressure readings, hypertension staging, lipid profile, smoking status, diabetes status and antihypertensive regimen were not abstracted from the medical records and were therefore unavailable for analysis; this constraint is addressed in Section 4.6. Monthly income was recorded but was not entered into the regression models because it was missing for 13 participants (4.6%) and because 58 participants (20.5%) reported no personal income, which makes the variable a poor proxy for household economic position; it is reported descriptively in Table 1.

**Table 1:** Sociodemographic and clinical characteristics of hypertensive patients attending two public hospitals in Addis Ababa, Ethiopia, February–August 2022 (*n* = 283).

Variable	Category	Frequency	%
Sex	Female	154	54.4
	Male	129	45.6
Age, years (mean ± SD 59.67 ± 13.76; range: 23–90)	< 50	66	23.3
	50–65	126	44.5
	> 65	91	32.2
Marital status	Married	136	48.1
	Widowed	64	22.6
	Divorced	60	21.2
	Single (never married)	23	8.1
Educational status	Unable to read and write	58	20.5
	Able to read and write, no formal schooling	29	10.2
	Primary school (Grades 1–8)	65	23.0
	Secondary school (Grades 9–10)	19	6.7
	Preparatory school (Grades 11–12)	36	12.7
	Above Grade 12	76	26.9
Religion	Orthodox	182	64.3
	Muslim	51	18.0
	Protestant	46	16.3
	Catholic	4	1.4
Monthly income, ETB (median: 2000; IQR: 450–5000)	No personal income	58	20.5
	1–2000	90	31.8
	2001–5000	67	23.7
	> 5000	55	19.4
	Not reported	13	4.6
Body mass index, kg/m^2^ (mean ± SD 25.48 ± 3.52)	Underweight (< 18.5)	2	0.7
	Normal (18.5–24.9)	132	46.6
	Overweight (25.0–29.9)	114	40.3
	Obese (≥ 30.0)	35	12.4
Family history of stroke	No	207	73.1
	Yes	76	26.9
Duration of hypertension, years (mean ± SD 10.51 ± 8.95; median: 8)	≤ 10	177	62.5
	11–20	70	24.7
	> 20	36	12.7

*Note:* Percentages are of the full sample (*n* = 283) and may not total 100 because of rounding. Monthly income was recorded but not entered into the regression models (Section 2.7).

Abbreviations: ETB, Ethiopian birr; IQR, interquartile range; SD, standard deviation.

### 2.8 Data Collection Instruments

A structured, interviewer‐administered questionnaire in three sections was used.

**Section I—Sociodemographic and clinical data.** Abstracted from the medical record and confirmed with the patient: age, sex, educational status, marital status, religion, monthly income, duration of hypertension, BMI, and family history of stroke.

**Section II—SPKQ.** A 32‐item instrument covering three domains: knowledge of stroke prevention measures (10 items), knowledge of warning signs (10 items) and knowledge of risk factors (12 items). Each item is answered yes or no, scored 1 for a correct response and 0 otherwise, giving a total of 0–32. The item content follows that used in previous Ethiopian studies of stroke prevention knowledge among hypertensive patients, which permits direct comparison [17, 18, 20].

**Section III—HPLP‐II.** A 52‐item instrument [26, 27] covering six domains: health responsibility (9 items), physical activity (8), nutrition (9), spiritual growth (9), interpersonal relations (9) and stress management (8). Items are rated on a 4‐point scale (1 = *never*, 2 = *sometimes*, 3 = *often*, 4 = *routinely*), giving a total of 52–208.

**Rationale for the HPLP‐II.** The HPLP‐II was selected because the behaviors that lower stroke risk in hypertensive patients are largely not stroke‐specific behaviors: they are the general health‐promoting behaviors of physical activity, dietary modification, stress management and engagement with health services, each of which appears as a distinct HPLP‐II domain and each of which is an established target of stroke primary prevention guidance [7, 8]. Using a validated general instrument also permits comparison with hypertensive and at‐risk populations studied elsewhere [28, 29], which a bespoke stroke‐practice checklist would not. This is nonetheless an indirect operationalisation: the HPLP‐II does not capture antihypertensive medication adherence, blood pressure self‐monitoring or intended response to warning signs, all of which are central to stroke prevention. This limitation is developed in Section 4.6.

### 2.9 Data Quality Assurance

The questionnaire was developed in English, translated into Amharic by a language expert and back‐translated into English to verify fidelity. It was pretested on 5% of the sample size among hypertensive patients at Tikur Anbessa Specialized Hospital, and items were amended for clarity before fieldwork. Two BSc nurses collected data after a half‐day training on study objectives and instrument administration. The principal investigator reviewed all completed questionnaires daily for completeness and internal consistency. Internal consistency of the instruments was additionally assessed in the present sample and is reported in Section 3.3.

### 2.10 Operational Definitions

**Knowledge regarding stroke prevention:** total SPKQ score across all 32 items, dichotomized at 60% of the maximum attainable score, rounded up to the next whole item:

- **Good knowledge:** ≥ 20 of 32 items correct (≥ 62.5%)
- **Poor knowledge:** ≤ 19 of 32 items correct.

The same rule was applied within each domain, giving thresholds of ≥ 6 of 10 for knowledge of prevention measures, ≥ 6 of 10 for knowledge of warning signs and ≥ 8 of 12 for knowledge of risk factors.

**Practice regarding stroke prevention:** total HPLP‐II score, interpreted against the published bands [30]: poor 52–90, moderate 91–129, good 130–168, excellent 169–208. The continuous score was used in all regression modeling; the bands are reported descriptively only.

### 2.11 Statistical Analysis

Data were entered and analyzed in IBM SPSS Statistics version 27 (IBM Corp., Armonk, NY, USA); all analyses were independently re‐run and verified in Python 3.12 using pandas 3.0, statsmodels 0.14 and SciPy 1.17. Continuous variables are summarized as mean ± standard deviation (SD) and categorical variables as frequency and percentage. Internal consistency was assessed with Cronbach's α.

**Model selection.** The two outcomes were modeled differently because they are measured on different scales. Knowledge was analyzed as a binary outcome because the SPKQ has an established competence threshold that carries a clinical interpretation (adequate vs. inadequate understanding), and because comparator Ethiopian studies report it dichotomously [17, 18, 20], preserving comparability. Practice was analyzed as a continuous outcome because the HPLP‐II summed score is an interval‐level measure whose full range carries information; dichotomizing it would have discarded variance and reduced power. Binary logistic regression was therefore used for knowledge and multivariable linear regression for practice.

**Variable selection.** Each candidate explanatory variable was first examined against the outcome in bivariable analysis, using the likelihood‐ratio test for the logistic models and the overall *F* test for the linear models. Variables with *p* < 0.20 were carried into the multivariable model. This threshold, rather than 0.05, is the conventional screening criterion for observational model building: a 0.05 screen is known to exclude variables that become important as confounders once other terms are present, and the 0.15–0.25 range is the established recommendation [31, 32]. All variables meeting the criterion were entered simultaneously; no stepwise selection was performed.

**Handling of sparse categories.** Two category collapses were specified before modeling to avoid separation and unstable estimates. “Unable to read and write” and “able to read and write without formal schooling” were combined into a single “no formal schooling” category, because no participant in the latter group met the knowledge threshold, which produced complete separation. The two underweight participants were combined with the normal‐weight group as BMI < 25 kg/m^2^. The four Catholic participants were combined with Protestant participants. Uncollapsed frequencies for all categories are given in Table 1.

**Assumption checking.** For the linear model, normality of the outcome and of the residuals was assessed by normal probability (P–P) plots and the Shapiro–Wilk test; homoscedasticity by inspection of residual‐versus‐predicted plots and the Breusch–Pagan test; independence of observations by study design and the Durbin–Watson statistic; and multicollinearity by variance inflation factor (VIF) and tolerance, taking VIF > 10 and tolerance < 0.10 as thresholds of concern. Influence was assessed with studentised residuals and Cook's distance. For the logistic model, calibration was assessed with the Hosmer–Lemeshow test and discrimination with the area under the receiver operating characteristic curve (AUC); multicollinearity was assessed by VIF on the design matrix.

**Inference.** All tests were two‐sided and *α* was set at 0.05. Associations are reported as crude and adjusted odds ratios (COR, AOR) with 95% confidence intervals (CI) for the knowledge model, and as unstandardized regression coefficients (*B*) with 95% CIs, together with standardized coefficients (β), for the practice model. The *p* values are reported as *p* < 0.001 below 0.001, to three decimal places between 0.001 and 0.01, to two decimal places at or above 0.01, and as *p* > 0.99 above 0.99.

**Pre‐specified versus exploratory analyses.** The knowledge and practice models described above were pre‐specified. Two sensitivity analyses, identified as such in the Results, were performed: the knowledge threshold was varied from ≥ 20 to ≥ 19 of 32, and the three knowledge domains were substituted for the overall knowledge variable, one at a time, in the practice model. No subgroup analyses were undertaken, and no interim or adaptive analyses were performed.

### 2.12 Ethical Considerations

Ethical clearance was granted by the Institutional Review Board of the Department of Nursing, College of Health Sciences, Addis Ababa University (reference CHS/NSG/341). Written permission was obtained from the administrations of SPHMMC, SPSH and Tikur Anbessa Specialized Hospital before data collection began. Written informed consent was obtained from every participant after explanation of the study's purpose, procedures, voluntary nature and the right to withdraw at any point without effect on their care. No personal identifiers were recorded; questionnaires were coded and stored securely, and access was restricted to the investigators. The study was conducted in accordance with the Declaration of Helsinki.

## 3 Results

### 3.1 Sociodemographic and Clinical Characteristics

Of 302 patients approached, 283 completed the interview, giving a response rate of 93.7% (Figure 1). Women made up 54.4% of the sample. Mean age was 59.67 ± 13.76 years (range: 23–90); 126 participants (44.5%) were aged 50–65 years, 91 (32.2%) were older than 65 and 66 (23.3%) were younger than 50. Just under half (48.1%) were married. Eighty‐seven participants (30.7%) had no formal schooling and 76 (26.9%) had studied beyond Grade 12. Mean BMI was 25.48 ± 3.52 kg/m^2^, and 134 participants (47.3%) had a BMI below 25 kg/m^2^. Seventy‐six participants (26.9%) reported a family history of stroke, and 177 (62.5%) had carried a hypertension diagnosis for 10 years or less (median: 8 years). Fifty‐eight participants (20.5%) reported no personal monthly income; the median among those who did was 2000 Ethiopian birr (Table 1).

### 3.2 Knowledge Regarding Stroke Prevention

The mean total SPKQ score was 12.87 ± 7.78 of a possible 32 (observed range: 0–29). Seventy‐seven participants (27.2%) answered at least 20 of the 32 items correctly and were classified as having good knowledge; 206 (72.8%) were classified as having poor knowledge. Within domains, 137 participants (48.4%) reached the threshold for knowledge of prevention measures (mean: 5.32 ± 1.87 of 10), 101 (35.7%) for warning signs (mean: 4.04 ± 3.85 of 10) and 59 (20.8%) for risk factors (mean: 3.51 ± 3.62 of 12) (Table 2).

**Table 2:** Participants' knowledge regarding stroke prevention, by item and domain (*n* = 283).

Item	Answered correctly, *n* (%)
Domain 1—Knowledge of stroke prevention measures (10 items)	
Antihypertensive medication adherence	258 (91.2)
Regular blood pressure check‐ups	253 (89.4)
Reduce intake of fatty food	242 (85.5)
Consume a low‐salt diet	220 (77.7)
Control blood sugar level	180 (63.6)
Maintain normal body weight	115 (40.6)
Eat fruit and vegetables regularly	114 (40.3)
Engage in regular physical activity	98 (34.6)
Avoid excessive alcohol use	15 (5.3)
Smoking abstinence	8 (2.8)
*Domain 1 score, mean ± *SD	*5.32* ± *1.87 of 10*
Good knowledge of prevention measures (≥ 6 of 10)	137 (48.4)
Domain 2—Knowledge of warning signs (10 items)	
Sudden loss or reduced sensation on one side of the body	164 (58.0)
Sudden weakness or paralysis on one side of the body	162 (57.2)
Sudden loss or reduced sensation all over the body	157 (55.5)
Sudden weakness or paralysis all over the body	157 (55.5)
Sudden dizziness, loss of balance or coordination	113 (39.9)
Sudden severe headache	93 (32.9)
Sudden difficulty speaking	87 (30.7)
Sudden loss of memory	86 (30.4)
Sudden difficulty swallowing	73 (25.8)
Sudden loss of vision	52 (18.4)
*Domain 2 score, mean ± *SD	*4.04* ± *3.85 of 10*
Good knowledge of warning signs (≥ 6 of 10)	101 (35.7)
Domain 3—Knowledge of risk factors (12 items)	
Advancing age	161 (56.9)
High blood pressure	123 (43.5)
Eating food containing too much fat	107 (37.8)
Excessive alcohol intake	107 (37.8)
Not exercising regularly	96 (33.9)
Being overweight or obese	95 (33.6)
Cigarette smoking	79 (27.9)
Diabetes mellitus	67 (23.7)
Heart disease	58 (20.5)
Family history of stroke	57 (20.1)
Polycythaemia	24 (8.5)
Use of oral contraceptives^a^	19 (7.0)
*Domain 3 score, mean ± *SD	*3.51* ± *3.62 of 12*
Good knowledge of risk factors (≥ 8 of 12)	59 (20.8)
*Overall SPKQ score, mean ± *SD	*12.87* ± *7.78 of 32*
Good overall knowledge of stroke prevention (≥ 20 of 32)	77 (27.2)

*Note:* The correct answer to every item is “yes”; one point was awarded for each correct response. Each domain and the overall score were dichotomized at 60% of the attainable score, rounded up to the next whole item.

Abbreviations: SD, standard deviation; SPKQ, Stroke Prevention Knowledge Questionnaire.

^a^ This item was not answered by 13 participants; the percentage is of the 270 who responded. For scoring, unanswered items were treated as incorrect; a sensitivity analysis excluding these participants is reported in Section 3.2.

Two pre‐specified sensitivity analyses were performed. Relaxing the overall threshold from ≥ 20 to ≥ 19 of 32 classified 80 participants (28.3%) as having good knowledge. Excluding the 13 participants for whom one risk‐factor item was missing left 77 of 270 (28.5%) classified as having good knowledge. Neither materially altered the finding.

Item‐level responses showed a consistent pattern. Recognition was high for the prevention measures that clinics actively deliver and monitor, antihypertensive medication adherence (91.2%), regular blood pressure checks (89.4%) and reduction of dietary fat (85.5%), and low for those that depend on patient‐initiated behavior, notably regular physical activity (34.6%) and regular consumption of fruit and vegetables (40.3%). Smoking abstinence (2.8%) and avoidance of excessive alcohol (5.3%) were almost never identified as preventive measures.

Among warning signs, the most frequently recognized were sudden loss or reduction of sensation on one side of the body (58.0%) and sudden unilateral weakness or paralysis (57.2%); the least recognized were sudden loss of vision (18.4%) and sudden difficulty swallowing (25.8%). Among risk factors, advancing age was identified by 161 participants (56.9%) and hypertension itself by only 123 (43.5%), while diabetes mellitus (23.7%), heart disease (20.5%) and family history of stroke (20.1%) were recognized by roughly one in five.

**Figure 2:**
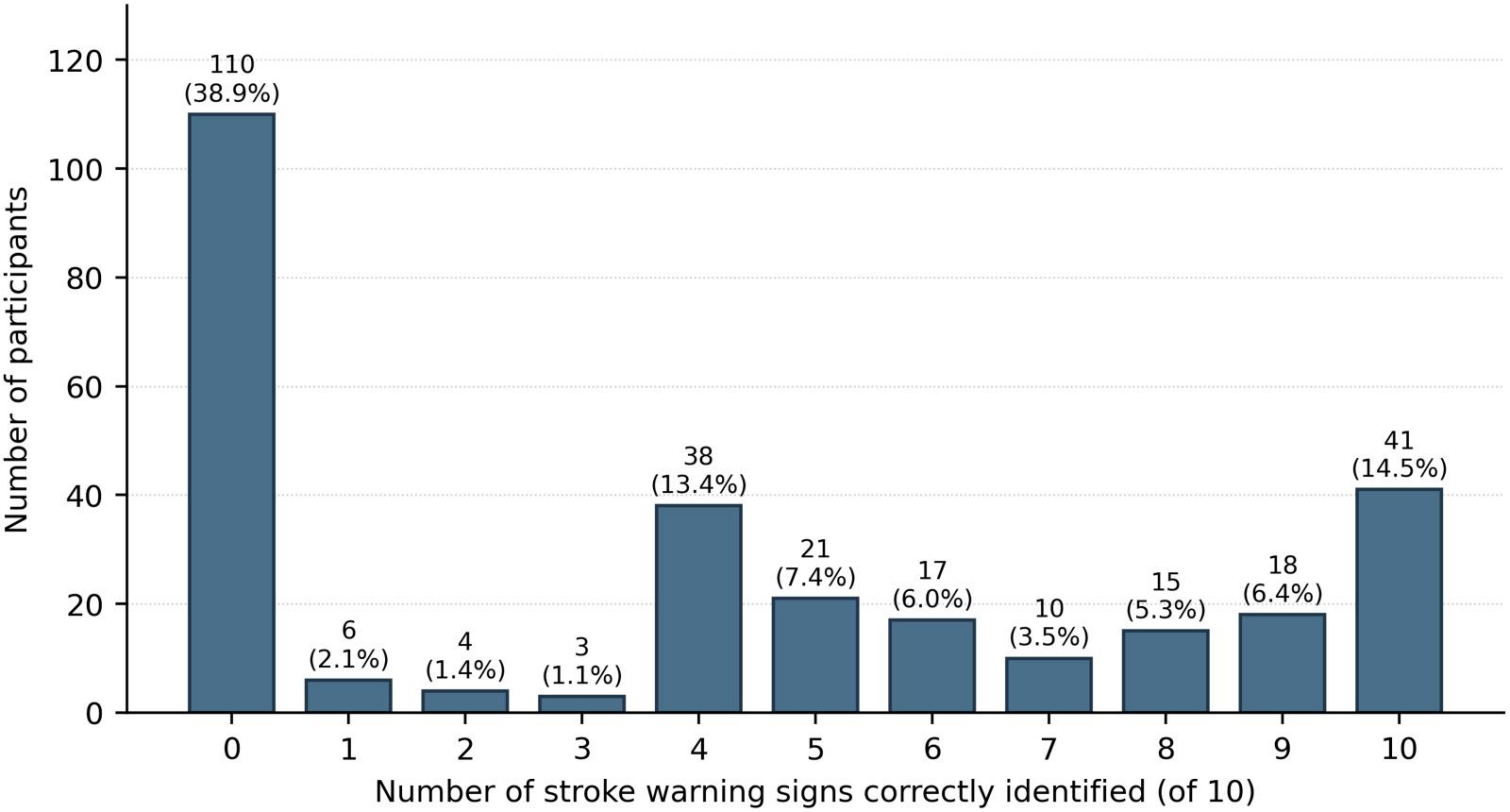
Number of stroke warning signs correctly identified, of a possible 10, by hypertensive patients attending two public hospitals in Addis Ababa, Ethiopia (*n* = 283). Bars show the number of participants; percentages of the sample are given above each bar.

A total of 110 participants (38.9%) could not name any warning sign of stroke, and 41 (14.5%) identified all 10 (Figure 2).

### 3.3 Practice Regarding Stroke Prevention

The mean total HPLP‐II score was 136.97 ± 26.71 (observed range: 78–193; median: 137), which falls within the “good” band (130–168). Domain means, in descending order, were interpersonal relations 30.08 ± 4.72, spiritual growth 25.64 ± 6.09, health responsibility 24.64 ± 6.55, stress management 20.64 ± 5.55, nutrition 19.65 ± 4.29 and physical activity 16.32 ± 5.20. Expressed as mean score per item, which allows domains of different length to be compared directly, physical activity was the weakest domain (2.04 of 4) and interpersonal relations the strongest (3.34 of 4) (Table 3).

**Table 3:** Health‐Promoting Lifestyle Profile II domain and total scores among hypertensive patients (*n* = 283).

Rank	Domain (items)	Mean ± SD	Mean score per item (of 4)	Cronbach's *α*
1	Interpersonal relations (9)	30.08 ± 4.72	3.34	0.769
2	Spiritual growth (9)	25.64 ± 6.09	2.85	0.784
3	Health responsibility (9)	24.64 ± 6.55	2.74	0.863
4	Stress management (8)	20.64 ± 5.55	2.58	0.786
5	Nutrition (9)	19.65 ± 4.29	2.18	0.682
6	Physical activity (8)	16.32 ± 5.20	2.04	0.853
	Overall HPLP‐II (52)	136.97 ± 26.71	2.63	0.945

*Note:* Possible range: 52–208 for the total and 1–4 per item. Observed total range: 78–193; median: 137. Bands: poor 8 (2.8%), moderate: 97 (34.3%), good: 139 (49.1%), excellent: 39 (13.8%). Domains are ranked by mean score per item, which permits comparison between domains of unequal length.

Abbreviations: HPLP‐II, Health‐Promoting Lifestyle Profile II; SD, standard deviation.

By band, 8 participants (2.8%) scored poor, 97 (34.3%) moderate, 139 (49.1%) good, and 39 (13.8%) excellent.

Internal consistency in this sample was high: Cronbach's *α* was 0.945 for the HPLP‐II total, with domain values ranging from 0.682 (nutrition) to 0.863 (health responsibility); for the SPKQ, *α* was 0.945 for the warning‐sign domain and 0.903 for the risk‐factor domain.

### 3.4 Factors Associated With Knowledge Regarding Stroke Prevention

All eight candidate variables (sex, age group, marital status, educational status, religion, BMI, family history of stroke, and duration of hypertension) met the *p* < 0.20 screening criterion in bivariable analysis and were entered simultaneously into the multivariable logistic model (Table 4). The model was informative overall (likelihood‐ratio *χ* ^2^ = 126.20, df = 17, *p* < 0.001; Nagelkerke *R* ^2^ = 0.522) and discriminated well (AUC: 0.887). Multicollinearity was not evident (maximum VIF: 2.90).

**Table 4:** Factors associated with good overall knowledge regarding stroke prevention: crude and adjusted binary logistic regression (*n* = 283).

Variable	Category	Poor, *n*	Good, *n*	COR (95% CI)	*p*	AOR (95% CI)	*p*
Sex	Female	121	33	1.00 (ref)	—	1.00 (ref)	—
	Male	85	44	1.90 (1.12–3.22)	0.02	1.18 (0.52–2.67)	0.69
Age, years	< 50	38	28	1.00 (ref)	—	1.00 (ref)	—
	50–65	90	36	0.54 (0.29–1.01)	0.05	0.35 (0.14–0.88)	0.03
	> 65	78	13	0.23 (0.11–0.49)	< 0.001	0.41 (0.12–1.39)	0.15
Marital status	Married	88	48	1.00 (ref)	—	1.00 (ref)	—
	Widowed	57	7	0.23 (0.10–0.53)	< 0.001	0.86 (0.25–2.97)	0.81
	Divorced	42	18	0.79 (0.41–1.51)	0.47	0.43 (0.17–1.10)	0.08
	Single (never married)	19	4	0.39 (0.12–1.20)	0.10	1.17 (0.21–6.34)	0.86
Educational status	No formal schooling	84	3	1.00 (ref)	—	1.00 (ref)	—
	Primary school (Grades 1–8)	56	9	4.50 (1.17–17.35)	0.03	5.44 (1.21–24.59)	0.03
	Secondary school (Grades 9–10)	16	3	5.25 (0.97–28.37)	0.05	6.07 (0.92–40.03)	0.06
	Preparatory school (Grades 11–12)	24	12	14.00 (3.65–53.68)	< 0.001	12.80 (2.60–63.00)	0.002
	Above Grade 12	26	50	53.85 (15.50–187.06)	< 0.001	50.43 (10.98–231.53)	< 0.001
Religion	Orthodox	135	47	1.00 (ref)	—	1.00 (ref)	—
	Muslim	47	4	0.24 (0.08–0.72)	0.01	0.27 (0.08–0.92)	0.04
	Protestant or Catholic	24	26	3.11 (1.63–5.94)	< 0.001	3.24 (1.29–8.13)	0.01
Body mass index, kg/m^2^	< 25	104	30	1.00 (ref)	—	1.00 (ref)	—
	25.0–29.9	76	38	1.73 (0.99–3.04)	0.06	1.21 (0.55–2.68)	0.63
	≥ 30.0	26	9	1.20 (0.51–2.84)	0.68	1.00 (0.31–3.21)	> 0.99
Family history of stroke	No	162	45	1.00 (ref)	—	1.00 (ref)	—
	Yes	44	32	2.62 (1.49–4.60)	< 0.001	2.76 (1.25–6.09)	0.01
Duration of hypertension, years	≤ 10	123	54	1.00 (ref)	—	1.00 (ref)	—
	11–20	50	20	0.91 (0.50–1.68)	0.76	1.51 (0.64–3.56)	0.35
	> 20	33	3	0.21 (0.06–0.70)	0.01	0.20 (0.04–1.00)	0.05

*Note:* Outcome is good overall knowledge, defined as ≥ 20 of 32 SPKQ items correct. All eight variables met the *p* < 0.20 bivariable screening criterion and were entered simultaneously; no stepwise selection was used. Model: likelihood‐ratio *χ* ^2^ = 126.20, df = 17, *p* < 0.001; Nagelkerke *R* ^2^ = 0.522; AUC: 0.887; maximum VIF: 2.90. All tests were two‐sided with *α* = 0.05. Category collapses are described in Section 2.11.

Abbreviations: AOR, adjusted odds ratio; CI, confidence interval; COR, crude odds ratio; ref, reference category.

Educational attainment showed a strong graded association with knowledge. Relative to participants with no formal schooling, the adjusted odds of good knowledge were higher among those educated beyond Grade 12 (AOR: 50.43, 95% CI: 10.98–231.53; *p* < 0.001), those who had completed preparatory school (AOR: 12.80, 95% CI: 2.60–63.00; *p* = 0.002) and those who had completed primary school (AOR: 5.44, 95% CI: 1.21–24.59; *p* = 0.03); the estimate for secondary school was of similar magnitude but did not reach significance (AOR: 6.07, 95% CI: 0.92–40.03; *p* = 0.06). The CIs are wide, reflecting the small number of participants with good knowledge in the lower attainment categories, and the point estimates should be read as indicating direction and gradient rather than precise magnitude.

Two further characteristics were independently associated with knowledge. Muslim participants had lower adjusted odds of good knowledge than Orthodox participants (AOR: 0.27, 95% CI: 0.08–0.92; *p* = 0.04) and Protestant or Catholic participants had higher odds (AOR: 3.24, 95% CI: 1.29–8.13; *p* = 0.01). Participants reporting a family history of stroke had higher adjusted odds of good knowledge than those without (AOR: 2.76, 95% CI: 1.25–6.09; *p* = 0.01).

Age showed a significant but non‐monotonic pattern: participants aged 50–65 years had lower adjusted odds than those under 50 (AOR: 0.35, 95% CI: 0.14–0.88; *p* = 0.03), whereas the estimate for those over 65, although lower in crude analysis, was not significant after adjustment (AOR: 0.41, 95% CI: 0.12–1.39; *p* = 0.15). The association with a hypertension duration exceeding 20 years was of borderline significance and its CI reached unity (AOR: 0.20, 95% CI: 0.04–1.00; *p* = 0.05). Sex, marital status and BMI were not independently associated with knowledge once education and the remaining covariates were accounted for.

The Hosmer–Lemeshow test was significant when calculated over 10 groups (*χ* ^2^ = 23.68, df = 8, *p* = 0.003) but not over five to eight groups (*p* = 0.30–0.41). Because every predictor in the model is categorical, predicted probabilities are heavily tied and decile boundaries fall arbitrarily within tied blocks; the discrepancy arose from two adjacent groups whose deviations were of opposite sign. Calibration should therefore be regarded as uncertain, and the model is presented as a description of adjusted associations rather than as a prediction tool.

### 3.5 Factors Associated With Practice Regarding Stroke Prevention

All linear regression assumptions were satisfied. Residuals were approximately normally distributed (Shapiro–Wilk *W* = 0.994, *p* = 0.27), variance was constant across fitted values (Breusch–Pagan LM = 26.88, *p* = 0.08), observations were independent (Durbin–Watson: 1.60), and multicollinearity was absent (maximum VIF: 2.94, mean: 1.63; minimum tolerance: 0.34). No observation was unduly influential (maximum absolute studentised residual: 2.50; maximum Cook's distance: 0.028).

All nine candidate variables met the *p* < 0.20 screening criterion and were entered simultaneously. The model explained just over half the variance in HPLP‐II score (*R* ^2^ = 0.530, adjusted *R* ^2^ = 0.498; *F*_(18, 264)_ = 16.52, *p* < 0.001). Three characteristics were independently associated with the practice score (Table 5).

- **Educational status.** Scores rose stepwise with attainment relative to participants with no formal schooling: primary school *B* = 13.29 (95% CI: 6.49–20.09), secondary school *B* = 24.59 (95% CI: 14.32–34.86), preparatory school *B* = 25.42 (95% CI: 16.71–34.13) and above Grade 12 *B* = 28.08 (95% CI: 19.73–36.43), all *p* < 0.001.
- **Marital status.** Widowed participants scored lower than married participants (*B* = −7.42, 95% CI: −14.43 to −0.41; *p* = 0.04). Divorced and never‐married participants did not differ significantly from married participants after adjustment.
- **Knowledge.** Participants with good overall knowledge scored 13.20 points higher on the HPLP‐II than those with poor knowledge (95% CI: 6.81–19.60; *p* < 0.001). Across the whole sample the total knowledge score and the HPLP‐II score were moderately correlated (Pearson *r* = 0.63, *p* < 0.001).

**Table 5:** Factors associated with practice regarding stroke prevention (total HPLP‐II score): crude and adjusted linear regression (*n* = 283).

Variable	Category	Crude *B* (95% CI)	*p*	Adjusted *B* (95% CI)	*β*	*p*
Sex	Female	Reference	—	Reference	—	—
	Male	11.50 (5.36 to 17.64)	< 0.001	3.62 (−1.86 to 9.10)	0.068	0.19
Age, years	< 50	Reference	—	Reference	—	—
	50–65	0.83 (−6.83 to 8.48)	0.83	1.06 (−5.65 to 7.76)	0.020	0.76
	> 65	−16.42 (−24.56 to −8.27)	< 0.001	−4.49 (−12.62 to 3.64)	−0.079	0.28
Marital status	Married	Reference	—	Reference	—	—
	Widowed	−27.24 (−34.43 to −20.04)	< 0.001	−7.42 (−14.43 to −0.41)	−0.116	0.04
	Divorced	−3.71 (−11.06 to 3.64)	0.32	−1.57 (−7.83 to 4.68)	−0.024	0.62
	Single (never married)	−23.95 (−34.65 to −13.25)	< 0.001	−8.09 (−18.38 to 2.21)	−0.083	0.12
Educational status	No formal schooling	Reference	—	Reference	—	—
	Primary school (Grades 1–8)	18.77 (12.36 to 25.17)	< 0.001	13.29 (6.49 to 20.09)	0.210	< 0.001
	Secondary school (Grades 9–10)	31.24 (21.35 to 41.13)	< 0.001	24.59 (14.32 to 34.86)	0.231	< 0.001
	Preparatory school (Grades 11–12)	36.88 (29.14 to 44.62)	< 0.001	25.42 (16.71 to 34.13)	0.318	< 0.001
	Above Grade 12	44.88 (38.75 to 51.01)	< 0.001	28.08 (19.73 to 36.43)	0.467	< 0.001
Religion	Orthodox	Reference	—	Reference	—	—
	Muslim	−7.28 (−15.33 to 0.78)	0.08	−2.33 (−8.79 to 4.13)	−0.034	0.48
	Protestant or Catholic	15.77 (7.65 to 23.88)	< 0.001	4.71 (−1.76 to 11.18)	0.067	0.15
Body mass index, kg/m^2^	< 25	Reference	—	Reference	—	—
	25.0–29.9	9.62 (3.00 to 16.24)	0.005	1.04 (−4.12 to 6.21)	0.019	0.69
	≥ 30.0	1.65 (−8.21 to 11.52)	0.74	−3.30 (−10.75 to 4.15)	−0.041	0.38
Family history of stroke	No	Reference	—	Reference	—	—
	Yes	7.69 (0.68 to 14.70)	0.03	1.71 (−3.61 to 7.03)	0.028	0.53
Duration of hypertension, years	≤ 10	Reference	—	Reference	—	—
	11–20	−2.24 (−9.61 to 5.13)	0.55	2.44 (−3.19 to 8.07)	0.040	0.39
	> 20	−12.06 (−21.60 to −2.51)	0.01	1.78 (−6.02 to 9.58)	0.022	0.65
Overall stroke prevention knowledge	Poor	Reference	—	Reference	—	—
	Good	30.87 (24.84 to 36.90)	< 0.001	13.20 (6.81 to 19.60)	0.220	< 0.001

*Note:* Outcome is the total HPLP‐II score (possible range: 52–208). All nine variables met the *p* < 0.20 bivariable screening criterion and were entered simultaneously. Model: *R* ^2^ = 0.530, adjusted *R* ^2^ = 0.498; *F*_(18, 264)_ = 16.52, *p* < 0.001. Assumption checks: Shapiro–Wilk *W* = 0.994, *p* = 0.27; Breusch–Pagan LM = 26.88, *p* = 0.08; Durbin–Watson: 1.60; maximum VIF: 2.94 (mean: 1.63), minimum tolerance: 0.34. Each of the three knowledge domains, substituted individually for the overall knowledge variable, was also independently associated with the outcome (Section 3.5); they were not entered together because the overall score is their sum. All tests were two‐sided with *α* = 0.05.

Abbreviations: β, standardized regression coefficient; B, unstandardized regression coefficient; CI, confidence interval; HPLP‐II, Health‐Promoting Lifestyle Profile II.

Sex, age group, religion, BMI, family history of stroke and duration of hypertension were each associated with the practice score in crude analysis but none remained independently associated after adjustment (Table 5). The crude associations with age (> 65 years, *B* = −16.42), sex (male: *B* = 11.50) and being widowed (*B* = −27.24) were substantially attenuated in the adjusted model, indicating that they were largely accounted for by educational attainment and by knowledge.

In a pre‐specified sensitivity analysis, each knowledge domain was substituted for the overall knowledge variable in turn, holding the covariate set constant. Each was independently associated with the practice score: prevention measures *B* = 20.34 (95% CI: 15.71–24.98; *p* < 0.001), warning signs *B* = 9.10 (95% CI: 3.29–14.90; *p* = 0.002) and risk factors *B* = 9.91 (95% CI: 3.44–16.37; *p* = 0.003). These three variables were not entered together, because the overall score is their sum and entering them simultaneously would induce collinearity by construction.

## 4 Discussion

### 4.1 Key Findings

Three findings distinguish this study from the Ethiopian literature that precedes it. First, the knowledge deficit is not a rural or peripheral phenomenon. These participants were attending specialist cardiac clinics at two federal teaching hospitals in the capital, with more frequent specialist contact than patients in the regional hospitals where comparable studies have been conducted, and yet only 27.2% reached the overall knowledge threshold, close to the 24.9% found at Debre Tabor General Hospital [17] and well below the 40.7% reported from the University of Gondar [20]. Access to specialist follow‐up does not, by itself, produce preventive knowledge.

Second, knowledge and reported practice diverge sharply in this population. Participants scored in the “good” band for health‐promoting lifestyle (136.97) while 72.8% failed the knowledge threshold and 38.9% could not name a single warning sign. Previous Ethiopian studies have measured one construct or the other; measuring both in the same participants shows that favorable aggregate lifestyle scores are not evidence of informed prevention. The two highest‐scoring HPLP‐II domains, interpersonal relations and spiritual growth, are not stroke‐protective behaviors, whereas the domain most directly relevant to stroke risk, physical activity, scored lowest of the six on a per‐item basis. Aggregate lifestyle scores can therefore mask precisely the deficit that matters clinically.

Third, the pattern of item‐level recognition locates the deficit. Participants readily identified the preventive measures that a follow‐up clinic delivers and monitors, medication adherence (91.2%) and blood pressure checks (89.4%), but not those requiring self‐directed change, such as regular physical activity (34.6%). Fewer than half (43.5%) identified hypertension itself as a stroke risk factor, despite every participant carrying that diagnosis. This is the pattern one would expect from clinical encounters that transmit instructions effectively but not the reasoning behind them.

### 4.2 Interpreting the Observed Associations

Educational attainment was the dominant correlate of both outcomes, with a clear gradient in each. Several mechanisms could plausibly generate this association, and the present design cannot distinguish between them: greater health literacy, allowing patients to extract more from a brief consultation; greater confidence in questioning clinicians; better access to written and online health material, most of which is not available in Amharic at accessible reading levels; and confounding by income and occupation, which correlate with education and independently shape food choice, time available for exercise and transport to appointments. The finding is consistent with reports from elsewhere in Ethiopia [18, 21, 33], Nigeria [22, 34] and Lebanon [35], and with the Viennese finding that knowledge about hypertension in stroke patients tracked educational level [36].

The most instructive feature of the adjusted models is what did not survive adjustment. Age, sex, religion, family history of stroke and duration of hypertension were each associated with the practice score in crude analysis, and the crude differences were large, participants over 65 scored 16.4 points lower than those under 50, and widowed participants 27.2 points lower than married participants, yet only widowhood remained independently associated once education and knowledge were in the model. The most economical reading is that much of what appears in unadjusted analyses as an effect of age, sex or religion on health‐promoting behavior in this population is, in this sample, a reflection of differences in educational attainment. Analyses that report crude associations alone, as several comparable studies have done, are likely to overstate the independent contribution of these characteristics.

Two characteristics were independently associated with knowledge but not with practice. Participants with a family history of stroke had nearly three times the adjusted odds of good knowledge, which is readily explained by direct exposure to the illness in a relative, but this additional knowledge was not accompanied by higher lifestyle scores. The association with religious affiliation is harder to interpret and we do not offer a doctrinal explanation for it: in this setting religion is closely bound to social network, neighborhood, migration history and schooling, and the Protestant and Catholic participants together numbered only 50, of whom 26 had good knowledge. We report the association because it was pre‐specified as a candidate variable and remained after adjustment, but we regard it as hypothesis‐generating.

The association between widowhood and lower practice scores is compatible with a household‐support mechanism, shared meal preparation, reminders about appointments and medication, and companionship during physical activity. It should be noted, however, that never‐married participants, who also lack a resident partner, did not differ significantly from married participants after adjustment, so a simple partnership‐support account does not fit the data cleanly, and bereavement itself may contribute.

Finally, participants with poor knowledge scored lower on practice, and this held for each knowledge domain separately. This is the association of greatest practical interest, but it is also the one where the cross‐sectional design is most limiting: knowledge may support behavior, behavior may generate knowledge through repeated clinical contact, or both may follow from education and socioeconomic position. The data cannot adjudicate between these, and we do not claim that improving knowledge would by itself improve practice.

### 4.3 Comparison With Previous Studies

Knowledge levels here were substantially lower than those reported from Sokoto, Nigeria (90.8%) [22], Vienna (77%) [36], and Kerala (78.3%) [37], and lower than the 42.7% for warning‐sign knowledge reported from Tikur Anbessa Specialized Hospital in this same city [33]. Direct comparison is limited by instrument heterogeneity: studies differ in whether they count spontaneously named warning signs, use recognition‐based checklists, or apply composite thresholds as we did, and the choice of threshold alone can move an estimate by tens of percentage points. Our own sensitivity analysis illustrates the point on a small scale, moving the threshold by a single item changed the estimate from 27.2% to 28.3%, and the differences between instruments are far larger than that. The comparison with Felege Hiwot Referral Hospital (18.3%) [18] is similarly constrained, since that study defined adequacy by the number of risk factors and warning signs named, as did a comparable Iraqi survey [38]. The recent report from a tertiary hospital in this city [19] is the closest available comparator and reached a similar conclusion about the size of the gap.

The HPLP‐II domain profile, with physical activity lowest, matches findings in hypertensive and general populations in Japan and the United States [29] and in studies relating the instrument to blood pressure [28]; physical inactivity is among the more consistently reported deficits across health‐promoting lifestyle studies. That the same domain ranks lowest here, in a population for whom activity is directly protective and for whom low‐cost interventions exist [39], identifies a target that is both modifiable and, at present, largely unaddressed in these clinics.

### 4.4 Currency of the Findings

Data were collected between February and August 2022, and two considerations bear on whether they remain representative. Hypertension prevalence and stroke burden in Ethiopia have not fallen since 2022 [12, 13], and no national program targeting stroke prevention literacy among hypertensive outpatients has been introduced in the interval, so there is no specific reason to expect the knowledge deficit to have narrowed. Against this, the collection period overlapped with continuing disruption to routine chronic‐disease services, which may have reduced the frequency of counseling contacts and depressed knowledge scores relative to a stable period. The findings should therefore be read as characterizing a structural gap rather than a precise contemporary point prevalence, and re‐measurement would be valuable.

### 4.5 Strengths

The study measured knowledge and practice concurrently in the same participants using two instruments, which permits the divergence between them to be observed directly rather than inferred across studies; both showed high internal consistency in this sample. Recruitment from two federal referral hospitals with national catchments produced a sociodemographically varied sample, and the 93.7% response rate limits non‐response bias within the attending population. The analysis was fully re‐run and independently verified against the raw data before submission, and the complete analysis specification, including the handling of sparse categories and the sensitivity analyses, was fixed before the models were fitted.

### 4.6 Limitations

Some limitations qualify these findings:

**Design.** The cross‐sectional design establishes associations only. No temporal ordering can be inferred between knowledge and practice, or between sociodemographic characteristics and either outcome, and none of the associations reported here should be read as causal.

**Self‐reported data.** Both instruments rely on self‐report. The HPLP‐II asks participants to characterize their own habitual behavior, which invites social desirability bias, particularly for diet, exercise and alcohol, where the desirable answer is obvious in a clinical setting, and recall bias for frequency‐based items. Interviewer administration, necessary given variable literacy, may have amplified this relative to self‐completion. Practice scores should therefore be regarded as an upper bound.

**Instrument fit.** The HPLP‐II is a general health‐promoting lifestyle measure, not a stroke prevention measure. It omits medication adherence, blood pressure self‐monitoring and intended response to warning signs, and it includes domains such as spiritual growth and interpersonal relations that carry little direct stroke‐preventive weight yet contribute equally to the total. A high total score is consequently a weak proxy for stroke‐preventive behavior, and the divergence we report between “good” practice scores and poor knowledge should be interpreted with this in mind.

**Sampling and generalizability.** Recruitment was hospital‐based at two federal tertiary centers, so patients who do not attend follow‐up, likely to be poorer, less educated and less informed, are systematically absent, and the true population‐level knowledge deficit is probably worse than reported. Both hospitals receive national referrals, which broadens the sample geographically but does not correct this selection.

**Unmeasured clinical confounding.** Blood pressure control, hypertension severity, comorbidities such as diabetes and dyslipidaemia, smoking status and antihypertensive regimen were not abstracted from the medical records and were unavailable for analysis. All are plausible confounders of the associations reported here, and their omission is the most important threat to the internal validity of the adjusted models. They could not be added retrospectively because the ethical approval covering record review has expired, and we recommend their inclusion in future work.

**Precision of the adjusted estimates.** Several categories contained few participants with good knowledge, which produced wide CIs in the logistic model, most conspicuously for the highest educational category. The direction and gradient of these associations are robust; their magnitudes are not precisely estimated.

**Exclusion of prior stroke and TIA.** Patients with previous cerebrovascular events were excluded, so these findings describe a primary‐prevention population and do not extend to secondary prevention, where knowledge is typically higher.

**Missing data.** One risk‐factor item was missing for 13 participants (4.6%) and monthly income for the same number; no other item was missing. Missing responses to the knowledge item were scored as incorrect, and a sensitivity analysis excluding these participants gave an essentially identical result (28.5% vs. 27.2%).

**Publication interval.** The interval between data collection in 2022 and publication limits currency, as discussed in Section 4.4.

## 5 Conclusion

Among hypertensive patients attending cardiac follow‐up at two public hospitals in Addis Ababa, knowledge regarding stroke prevention was poor: fewer than 3 in 10 met the overall competence threshold, only about a third recognized warning signs adequately, and nearly two in five could not name a single warning sign. Health‐promoting lifestyle scores were nevertheless in the good range, driven by domains with limited stroke‐preventive relevance, while physical activity, the domain most directly protective, scored lowest.

Educational attainment was associated with both outcomes and accounted for most of the crude associations with age, sex and religion, and lower knowledge was associated with lower practice scores across every knowledge domain. Because the design is cross‐sectional, these are associations rather than causal relationships.

The practical implication is narrow but firm: attendance at a specialist clinic cannot be taken as evidence that a patient understands why the treatment matters. Prevention counseling in these settings should verify comprehension of warning signs and risk factors rather than assume it, should be delivered in formats accessible to patients with limited formal education, and should give specific attention to physical activity.

### 5.1 Recommendations

- **Verify rather than assume comprehension:** cardiac follow‐up clinics should incorporate a brief check of warning‐sign and risk‐factor recognition into routine review, since 38.9% of attending patients could name no warning sign at all and fewer than half identified hypertension as a stroke risk factor.
- **Adapt materials to educational level**: given the graded association with education, counseling materials should be available in pictorial and spoken Amharic formats that do not depend on reading fluency.
- **Target physical activity specifically:** physical activity was the weakest HPLP‐II domain on a per‐item basis and was identified as a preventive measure by only 34.6% of participants, yet it is directly modifiable. Structured, low‐cost activity advice appropriate to older patients should be part of routine counseling.
- **Extend counseling to accompanying family members:** given the lower practice scores among widowed participants and the plausible role of household support.
- **Collect the clinical variables that were missing here:** blood pressure control, hypertension stage, comorbidity and treatment regimen, in future studies of this question, so that the associations reported here can be tested with adequate adjustment.

## Author Contributions

**Hana Shafi Amde:** conceptualization, investigation, funding acquisition, writing – original draft, writing – review and editing, methodology, data curation, project administration. **Alfoalem Araba Abiye:** investigation, writing – original draft, writing – review and editing, methodology, software, formal analysis, data curation, visualization. **Negalign Getahun Dinegde:** supervision, resources, validation, writing – review and editing. **Emebet Berhane Woldemariam:** supervision, writing – review and editing, validation, methodology, conceptualization.

## Funding

The authors have nothing to report.

## Ethics Statement

Ethical clearance was obtained from the Institutional Review Board of the Department of Nursing, College of Health Sciences, Addis Ababa University (reference CHS/NSG/341). Written permission was obtained from the administrations of St. Paul's Hospital Millennium Medical College, St. Peter's Specialized Hospital and Tikur Anbessa Specialized Hospital. The study was conducted in accordance with the Declaration of Helsinki.

## Consent

Written informed consent was obtained from all participants after explanation of the purpose, procedures, and voluntary nature of the study and of their right to withdraw at any time without effect on their care.

## Conflicts of Interest

The authors declare no conflicts of interest.

## Transparency Statement

Hana Shafi Amde affirms that this manuscript is an honest, accurate and transparent account of the study being reported; that no important aspects of the study have been omitted; and that any discrepancies from the study as planned have been explained.

## Supporting information


Supporting File

## Data Availability

The data that support the findings of this study are available from the corresponding author upon reasonable request. The data are not publicly available because they contain information that could compromise the privacy of research participants.
