# Supplementary material for: Knowledge and Practice Regarding Stroke Prevention and Associated Factors Among Hypertensive Patients at Selected Public Hospitals in Addis Ababa, Ethiopia: A Cross‐Sectional Study
Source: Health Sci Rep. 2026 Sep 17;9(9):e73268. doi: 10.1002/hsr2.73268 (PMC13586444; doi:10.1002/hsr2.73268)
Supplement: Supplementary file 1 [file HSR2-9-e73268-s001.pdf]

Supplementary File 1

STROBE Statement — checklist of items that should be included in reports of cross-sectional studies

Manuscript: Knowledge and Practice Regarding Stroke Prevention and Associated Factors among Hypertensive Patients at Selected Public Hospitals in Addis Ababa, Ethiopia: A Cross-Sectional Study

Journal: Health Science Reports (Wiley). Manuscript ID: 1625207.

Section, table and figure references refer to the accepted manuscript.

No.	Item	Recommendation	Reported in (section / table / figure)
1	**Title and abstract**	(a) Indicate the study's design with a commonly used term in the title or the abstract	Title ("A Cross-Sectional Study"); Abstract, Methods
		(b) Provide in the abstract an informative and balanced summary of what was done and what was found	Abstract (298 words, structured as Background and Aims / Methods / Results / Conclusion)
**Introduction**			
2	**Background/rationale**	Explain the scientific background and rationale for the investigation being reported	Section 1, paragraphs 1–3
3	**Objectives**	State specific objectives, including any prespecified hypotheses	Section 1, final paragraph (three numbered objectives; objective iii identified as co-primary and analytic)
**Methods**			
4	**Study design**	Present key elements of study design early in the paper	Section 2 preamble; Section 2.2
5	**Setting**	Describe the setting, locations, and relevant dates, including periods of recruitment, exposure, follow-up, and data collection	Sections 2.1 and 2.2 (two federal teaching hospitals, Addis Ababa; 1 February to 20 August 2022)
6	**Participants**	(a) Give the eligibility criteria, and the sources and methods of selection of participants	Sections 2.3, 2.4 and 2.6; Figure 1
7	**Variables**	Clearly define all outcomes, exposures, predictors, potential confounders, and effect modifiers. Give diagnostic criteria, if applicable	Sections 2.7 and 2.10 (measurement scale of each outcome stated; thresholds and their derivation stated)
8	**Data sources / measurement**	For each variable of interest, give sources of data and details of methods of assessment (measurement). Describe comparability of assessment methods if there is more than one group	Section 2.8 (instrument descriptions, HPLP-II rationale, source publications cited); Section 2.9
9	**Bias**	Describe any efforts to address potential sources of bias	Sections 2.9 (translation, back-translation, pretest, daily review) and 4.6 (self-report, social desirability, interviewer administration, hospital-based sampling, unmeasured confounding — each with direction of likely bias)

No.	Item	Recommendation	Reported in (section / table / figure)
10	**Study size**	Explain how the study size was arrived at	Section 2.5 (single population proportion formula, parameters stated); Figure 1
11	**Quantitative variables**	Explain how quantitative variables were handled in the analyses. If applicable, describe which groupings were chosen and why	Sections 2.10 and 2.11 ("Model selection", "Handling of sparse categories"); knowledge dichotomised with the rule stated, HPLP-II retained continuous with the rationale stated
12	**Statistical methods**	(a) Describe all statistical methods, including those used to control for confounding	Section 2.11
		(b) Describe any methods used to examine subgroups and interactions	Section 2.11 ("Pre-specified versus exploratory analyses") — no subgroup or interaction analyses were performed
		(c) Explain how missing data were addressed	Section 2.11; Section 3.2 (sensitivity analysis); Section 4.6 ("Missing data")
		(d) If applicable, describe analytical methods taking account of sampling strategy	Section 2.6 (proportional allocation); no design weights were applied, as allocation was proportional to caseload
		(e) Describe any sensitivity analyses	Section 2.11; Sections 3.2 and 3.5 (two pre-specified sensitivity analyses)
**Results**			
13	**Participants**	(a) Report numbers of individuals at each stage of the study	Section 3.1; Figure 1 (302 approached, 283 completed)
		(b) Give reasons for non-participation at each stage	Figure 1 (19 non-participants: declined consent or unable to complete the interview)
		(c) Consider use of a flow diagram	Figure 1
14	**Descriptive data**	(a) Give characteristics of study participants and information on exposures and potential confounders	Section 3.1; Table 1
		(b) Indicate the number of participants with missing data for each variable of interest	Table 1 (income, 13); Table 2 note (one risk-factor item, 13); no other missing data
15	**Outcome data**	Report numbers of outcome events or summary measures	Sections 3.2 and 3.3; Tables 2 and 3; Figure 2
16	**Main results**	(a) Give unadjusted estimates and, if applicable, confounder-adjusted estimates and their precision. Make clear which confounders were adjusted for and why they were included	Tables 4 and 5 (crude and adjusted estimates with 95% CIs for every category); Section 2.11 ("Variable selection")
		(b) Report category boundaries when continuous variables were categorized	Sections 2.10 and 3.1; Tables 1, 4 and 5

No.	Item	Recommendation	Reported in (section / table / figure)
		(c) If relevant, consider translating estimates of relative risk into absolute risk for a meaningful time period	Not applicable (no time period; prevalence and mean differences are reported on the absolute scale in Tables 2, 3 and 5)
17	**Other analyses**	Report other analyses done — eg analyses of subgroups and interactions, and sensitivity analyses	Sections 3.2 and 3.5 (both sensitivity analyses reported with their results); no subgroup or interaction analyses
**Discussion**			
18	**Key results**	Summarise key results with reference to study objectives	Section 4.1; Section 5
19	**Limitations**	Discuss limitations of the study, taking into account sources of potential bias or imprecision. Discuss both direction and magnitude of any potential bias	Section 4.6 (nine named limitations, each with the direction of likely bias stated)
20	**Interpretation**	Give a cautious overall interpretation of results considering objectives, limitations, multiplicity of analyses, results from similar studies, and other relevant evidence	Sections 4.1–4.4; causal language removed throughout (Section 4.2, final paragraph)
21	**Generalisability**	Discuss the generalisability (external validity) of the study results	Section 4.4 ("Currency of the findings"); Section 4.6 ("Sampling and generalisability", "Exclusion of prior stroke and TIA")
**Other information**			
22	**Funding**	Give the source of funding and the role of the funders for the present study and, if applicable, for the original study on which the present article is based	Declarations, "Funding" (no specific grant received)

Note: An Explanation and Elaboration article discusses each checklist item and gives methodological background and published examples of transparent reporting. The STROBE checklist is best used in conjunction with this article (freely available on the websites of PLoS Medicine at http://www.plosmedicine.org/, Annals of Internal Medicine at http://www.annals.org/, and Epidemiology at http://www.epidem.com/). Information on the STROBE Initiative is available at http://www.strobe-statement.org.
